# Temporal Dynamics of Catestatin Across Gestation: Links to Metabolic and Hemodynamic Adaptation

**DOI:** 10.3390/life16060896

**Published:** 2026-05-27

**Authors:** Zdenka Sunjic Lovric, Jasminka Resic Karara, Vedran Stefanovic, Bianka Mimica, Marko Kumric, Daniela Supe-Domic, Roko Santic, Josko Bozic

**Affiliations:** 1 Department of Gynecology and Obstetrics, University Hospital of Split, Spinciceva 1, 21000 Split, Croatia; zsunjic@kbsplit.hr (Z.S.L.); jreskara@kbsplit.hr (J.R.K.); bmimica@kbsplit.hr (B.M.); 2 Department of Health Studies, University of Split, Rudera Boskovica 35, 21000 Split, Croatia; dsupe@kbsplit.hr; 3 Department of Obstetrics and Gynecology, University of Helsinki and Helsinki University Hospital, 00029 Helsinki, Finland; vedran.stefanovic@hus.fi; 4 Department of Cardiovascular Diseases, University Hospital of Split, Spinciceva 1, 21000 Split, Croatia; marko.kumric@mefst.hr; 5 Laboratory for Cardiometabolic Research, School of Medicine, University of Split, Soltanska 2A, 21000 Split, Croatia; roko.santic@mefst.hr; 6 Department of Pathophysiology, School of Medicine, University of Split, Soltanska 2A, 21000 Split, Croatia; 7 Laboratory Diagnostics, Department of Medical, University Hospital of Split, Spinciceva 1, 21000 Split, Croatia

**Keywords:** catestatin, pregnancy, blood pressure, hypertensive disorders, biomarker

## Abstract

Catestatin is a chromogranin A–derived peptide involved in sympathetic, cardiovascular, inflammatory, and metabolic regulation, but its longitudinal profile during pregnancy remains insufficiently defined. This prospective cohort study aimed to evaluate changes in serum catestatin concentrations from the first to the third trimester and to explore their associations with blood pressure and metabolic parameters in initially low-risk singleton pregnancies. Fifty pregnant women were followed longitudinally from 11–13 + 6/7 to 30–41 + 5/7weeks of gestation. Clinical and biochemical parameters were assessed at both visits, and serum catestatin concentrations were measured using a commercial enzyme immunoassay. Serum catestatin concentrations were significantly lower in the third trimester than in the first trimester (median [IQR]: 9.4 [4.9–15.5] vs. 13.4 [9.9–24.6] ng/mL; *p* < 0.001). Longitudinal changes in catestatin were positively associated with third-trimester insulin concentrations after adjustment for selected covariates. Third-trimester catestatin concentrations were positively correlated with systolic blood pressure (r = 0.356, *p* = 0.011) and remained associated with systolic blood pressure in a limited multivariable model. These findings suggest that catestatin concentrations decline from early to late pregnancy and may reflect selected metabolic and hemodynamic changes. Larger longitudinal studies including pathological pregnancy cohorts are needed to clarify its clinical relevance.

## 1. Introduction

Pregnancy involves significant physiological adaptations, particularly within the cardiovascular system in order to support fetal development. It is well established that blood pressure declines during pregnancy, with the nadir typically occurring between 22 and 24 weeks of gestation. This reduction is primarily driven by a significant drop in peripheral vascular resistance due to systemic vasodilation. The decrease in peripheral resistance has been reported to range from 35% to 40% [1,2]. After reaching its lowest point in mid-gestation, blood pressure gradually rises during the later stages of pregnancy [3].

One factor implicated in regulating these changes is catestatin (CST), a bioactive peptide derived from chromogranin A (CgA). CST plays a central role in cardiovascular homeostasis by modulating sympathetic nervous system (SNS) activity and regulating blood pressure. It functions as an endogenous inhibitor of catecholamine release by binding to nicotinic acetylcholine receptors on chromaffin cells, thereby attenuating excessive SNS activation [4,5]. CST also exerts antihypertensive, anti-inflammatory, and cardioprotective effects by promoting vasodilation, enhancing endothelial function, and reducing oxidative stress [4,6,7,8].

While the role of CST in SNS regulation and blood pressure control is well-documented in non-pregnant adults [5,9,10], its role during pregnancy remains incompletely understood. Limited evidence suggests that CST levels may rise during gestation, but the relationship between CST and blood pressure-especially in conditions like preeclampsia has not been fully elucidated, and the available evidence remains controversial [11,12,13,14]. In placental tissue, Bralewska et al. reported increased chromogranin A gene expression in pregnancies complicated by preeclampsia compared with normotensive pregnancies [15]. In contrast, CST protein concentrations were reduced in preeclamptic samples, whereas CgA protein levels did not differ significantly between groups.

Palmrich et al. demonstrated CST levels were significantly decreased in women with preeclampsia compared to healthy controls indicating an association between decreased CST values and the development of preeclampsia [11]. There was no significant difference in catestatin values between early-onset preeclampsia and late-onset preeclampsia. Of importance, modelling the occurrence of preeclampsia via logistic regression was improved when adding CST as a predictive factor. However, the role of CST in pregnancy-related vascular adaptations, including changes in sympathetic tone and vascular reactivity, remains underexplored. Gestational hypertension and preeclampsia represent clinically important hypertensive disorders of pregnancy and remain key causes of adverse maternal and fetal outcomes, often in association with heightened sympathetic activation and endothelial dysfunction [16]. Emerging evidence suggests that CST levels may be altered in these conditions, potentially reflecting a compensatory response to SNS overactivity and vascular dysregulation. However, the precise role of CST in pregnancy-induced hypertension remains unclear.

The present study aimed to characterize longitudinal changes in serum CST concentrations from the first to the third trimester in initially low-risk singleton pregnancies and to explore their associations with blood pressure and selected metabolic parameters. The study was not designed to compare established pathological pregnancy phenotypes, such as preeclampsia, chronic hypertension, or pregestational diabetes, but rather to provide physiological longitudinal data on CST during pregnancy. Given CST’s pleiotropic effects, this research seeks to clarify its role in physiological adaptations during pregnancy.

## 2. Materials and Methods

### 2.1. Study Design and Ethical Considerations

This longitudinal prospective study was conducted at the University Hospital of Split, Croatia, between September 2022 and September 2023 to investigate the dynamics of catestatin during pregnancy and its association with arterial blood pressure. Ethical approval was obtained from the Ethics Committee of the University Hospital of Split (Class: 500-03/22-01/120, Number: 2181-147/01/06/M.S.-22-03) (29 June 2022), and the study adhered to the principles of the Declaration of Helsinki (2013 revision). Informed written consent was obtained from all pregnant participants after they received comprehensive information about the study’s purpose and procedures.

### 2.2. Subjects and Inclusion/Exclusion Criteria

Fifty pregnant women were followed longitudinally from the first to the third trimester, representing a continuation of our previous cross-sectional study that assessed serum catestatin concentrations in 72 healthy pregnant women during the first trimester and compared them with those of 57 healthy non-pregnant controls. A total of 50 pregnant women who underwent early combined screening for chromosomal abnormalities (11–13 + 6/7 weeks’ gestation) at the Department of Obstetrics and Gynecology, University Hospital Center Split, were enrolled. Eligible participants were women aged 18–40 years with a singleton pregnancy who attended first-trimester screening at 11–13 + 6/7 weeks of gestation, irrespective of parity.

Exclusion criteria were: recurrent miscarriages, known hypertensive disorders, pregestational diabetes, pregnancies via egg donation, fetal congenital anomalies, and multiple pregnancies. These criteria were applied to define an initially low-risk cohort. Therefore, women with chronic hypertension, known hypertensive disorders, or pregestational diabetes were not included at recruitment and could not be analyzed as baseline disease groups. None of the participants used acetylsalicylic acid or any other permanent medication.

Prior to the study commencement, a sample size analysis was conducted using the statistical software MedCalc (MedCalc Software, Ostend, Belgium, version 20.113), based on pilot study data involving 10 pregnant women and 10 healthy controls. Serum catestatin levels, as the primary study outcome, were used to determine the required sample size. Preliminary estimation indicated that, to achieve a statistical power of 90% with a type I error rate (α) of 0.05, at least 50 participants per group were needed. This sample size was considered sufficient for the primary longitudinal assessment of serum catestatin concentrations, but not for complication-specific subgroup comparisons.

### 2.3. Clinical and Laboratory Evaluations

Clinical data were collected during the mini anomaly scan (11–13 + 6/7 weeks’ gestation), including gravidity, parity, gestational age, height, body mass, BMI, medical history, arterial blood pressure, uterine artery pulsatility index (PI), crown-rump length (CRL), and biparietal diameter. Uterine artery PI was measured using Doppler ultrasound (Voluson E10), and values exceeding the 95th percentile were considered elevated [17].

Following ultrasound assessment, participants were referred for laboratory testing at the Department of Medical Laboratory Diagnostics, University Hospital of Split. Height was measured using a stadiometer (Seca, Birmingham, UK), and body mass with a bioelectrical impedance scale (Tanita DC-360 S, Tanita, Tokyo, Japan). BMI was calculated as weight (kg) divided by height squared (m^2^). Blood pressure was measured in accordance with European Society of Cardiology recommendations using a mercury sphygmomanometer and the Riva-Rocci method. Measurements were performed after at least 5 min of seated rest, with the participant’s arm supported at heart level and using an appropriately sized cuff. At each study visit, two blood pressure readings were obtained 1–2 min apart, and the average value was used for analysis. If the two readings differed substantially, an additional measurement was performed and the average of the closest two readings was recorded. The same examiner performed all blood pressure measurements at both the first- and third-trimester visits, ensuring consistency across the study.

The mini anomaly scan also included risk assessment for chromosomal abnormalities by integrating maternal characteristics (primarily maternal age), biochemical markers (free ß-hCG and PAPP-A), and ultrasound indicators such as nuchal translucency, nasal bone, ductus venosus flow, and fetal structural anomalies. Gestational age was confirmed by last menstrual period and CRL measurement.

After an overnight fast, approximately 15 mL of venous blood was drawn by an experienced technician. Immediate analyses included total cholesterol, triglycerides, HDL and LDL cholesterol, hemoglobin, and fasting plasma glucose. Serum samples were stored at −80 °C for later measurement of catestatin levels using a commercially available enzyme immunoassay (EIA) kit (Phoenix Pharmaceuticals Inc., Burlingame, CA, USA; Catalog #: EK-053-27CE). The reported assay sensitivity was 0.05 ng/mL, with 100% cross-reactivity for endogenous human catestatin. The intra-assay and inter-assay coefficients of variation were <10% and <15%, respectively. According to the manufacturer’s documentation, the linear range was 0–100 ng/mL. Samples were analyzed according to the manufacturer’s protocol, and final concentrations were calculated after accounting for the applied dilution factor.

Participants were re-evaluated in the third trimester, at 30–41 + 5/7. Clinical and biochemical parameters were reassessed to track dynamic changes throughout pregnancy. Blood pressure was re-measured using the same protocol as in the first trimester. Obstetric records were reviewed for newly diagnosed pregnancy complications during follow-up, including gestational hypertension, preeclampsia, and gestational diabetes mellitus, when present. All blood pressure measurements in both the initial cohort of 72 women and the final longitudinal sample of 50 women were performed by the same examiner, ensuring consistency across the study. Although the cohort was recruited as an initially low-risk singleton pregnancy cohort, incident pregnancy complications diagnosed during routine antenatal follow-up were recorded from available obstetric medical records. Hypertensive disorders of pregnancy were classified according to ISSHP criteria, while gestational diabetes mellitus was diagnosed according to the institutional antenatal care protocol. Chronic hypertension and pregestational diabetes were exclusion criteria at recruitment. Given the exploratory design and limited sample size, pregnancy complications were not treated as predefined outcome groups for subgroup comparison, but were considered descriptively and, where appropriate, as adjustment variables in multivariable analyses.

Blood samples were obtained and processed in the same manner as during the first trimester. All analyses were conducted in a certified institutional biochemical laboratory by an experienced biochemist blinded to participants’ clinical characteristics, in accordance with standard operating procedures.

### 2.4. Statistical Analysis

All statistical analyses were performed using SPSS software (version 29.0, IBM, Chicago, IL, USA), while graphical representations were created using Prism 6 for Windows^®^ (version 9.4.1, GraphPad, La Jolla, CA, USA). Quantitative data are presented as mean ± standard deviation (SD) or median with interquartile range, depending on data distribution characteristics. The normality of data distribution was assessed using the Shapiro–Wilk test. Comparisons of quantitative variables were conducted using Student’s *t*-test for normally distributed data or the Mann–Whitney U test for non-normally distributed data. Categorical data were compared using the chi-square test, with results expressed as count (n) and percentage (%). Spearman’s rank correlation was used to examine associations between serum catestatin levels and clinical and laboratory parameters. To determine whether catestatin concentrations were independently associated with selected clinical values, multiple linear regression analysis was applied. In the model evaluating Δ catestatin, gestational diabetes mellitus status was included as an adjustment covariate because insulin metabolism was one of the prespecified metabolic domains of interest. However, because the cohort was initially low risk and was not designed or powered for complication-specific subgroup analyses, formal comparisons of catestatin concentrations according to preeclampsia, chronic hypertension, pregestational diabetes, or gestational diabetes mellitus were not performed. Statistical significance was set at *p* < 0.05.

## 3. Results

Baseline characteristics of the studied population are presented in Table 1. The first-trimester assessment was performed at 11–13 + 6/7 weeks of gestation, while the third-trimester follow-up was performed at 30–41 + 5/7 weeks of gestation. Mean age of women was 31.6 ± 4.2, with an median BMI was 22.7 kg/m^2^ [IQR 21.1–25.3] and systolic blood pressure of 124.8 ± 15.3 mmHg. Seven (14%) of women had abnormal uterine artery PI and 5 (10%) had history of miscarriage.

**Figure 1:**
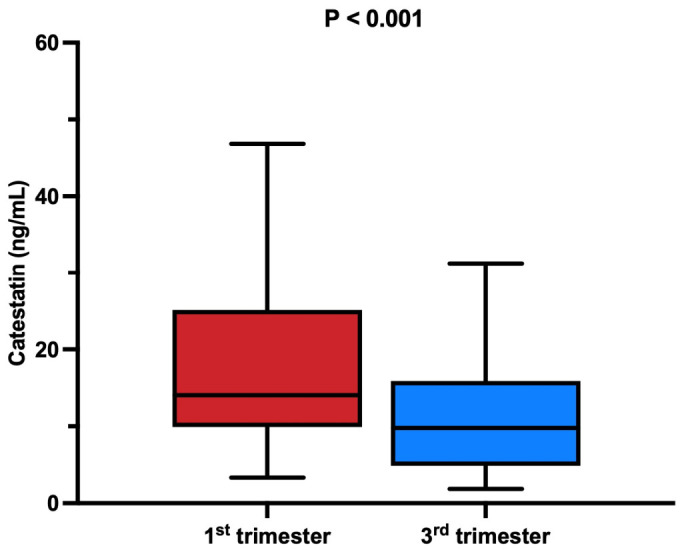
Comparison of catestatin levels between first and third trimester. Box plots show serum catestatin concentrations in the first and third trimesters. The central line represents the median, boxes indicate the interquartile range, and individual points denote outliers. Serum catestatin concentrations were significantly lower in the third trimester than in the first trimester (median [IQR]: 9.4 [4.9–15.5] vs. 13.4 [9.9–24.6] ng/mL; *p* < 0.001, Wilcoxon signed-rank test).

Serum catestatin concentrations were significantly lower in the third trimester in comparison with the first trimester of pregnancy (median [IQR]: 9.4 [4.9–15.5] vs. 13.4 [9.9–24.6] ng/mL; *p* < 0.001) (Figure 1), with a large effect size (r = 0.61, 95% CI 0.36–0.78).

Furthermore, a series of univariate analyses was performed to examine the associations between changes in catestatin levels across trimesters (Δ Catestatin) and relevant clinical and laboratory parameters (Table 2).

Δ Catestatin demonstrated a positive association with third-trimester insulin concentrations. This relationship remained significant in multivariate analysis after adjustment for gestational diabetes status, changes in systolic blood pressure, and serum creatinine levels (Table 3).

**Figure 2:**
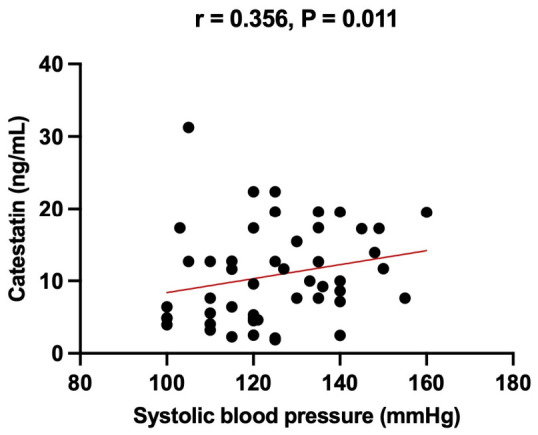
Association between third-trimester serum catestatin concentrations and systolic blood pressure. Each point represents an individual participant, and the red line indicates the fitted trend. Serum catestatin levels were positively correlated with systolic blood pressure in the third trimester, as assessed by Spearman’s rank correlation analysis (*r* = 0.356, *p* = 0.011).

Serum catestatin levels in the third trimester were positively associated with systolic blood pressure in the same trimester (r = 0.356, *p* = 0.011) (Figure 2).

This association remained significant in multivariate analysis after adjustment for age, insulin, and serum creatinine levels (Table 4).

## 4. Discussion

### 4.1. Summary of Main Findings

To the best of our knowledge, this is the first longitudinal study to characterise serum catestatin (CST) dynamics across human pregnancy. Building on our previous cross-sectional observation that first-trimester CST is elevated relative to non-pregnant women [18], the present cohort demonstrates a significant decline in CST concentrations from the first to the third trimester at the group level, with limited individual variability. Two associations emerged in the third trimester: a positive correlation between serum CST and systolic blood pressure, and an positive association between the longitudinal change in CST (Δ catestatin) and third-trimester insulin concentrations, the latter persisting in multivariable models. No association was observed between Δ CST and the change in blood pressure across gestation. These findings should be interpreted in the context of the small, single-centre, ethnically homogeneous sample and the absence of pregnancy-specific reference intervals for CST [19].

### 4.2. Comparison with Existing Human Pregnancy Data

Direct longitudinal data on circulating CST in human pregnancy are essentially absent, and the available cross-sectional studies are heterogeneous in design, gestational sampling and clinical phenotype. In normotensive pregnancy, our group previously reported elevated first-trimester CST relative to non-pregnant women [18]; the present cohort extends this observation by showing that, after the higher first-trimester concentrations observed in our previous work, CST declined toward the third trimester. This biphasic pattern is consistent with the limited human data suggesting that CST changes during gestation are dynamic rather than monotonic, and that single time-point measurements are insufficient to characterise its trajectory.

In hypertensive disorders of pregnancy, the literature remains discordant. Palmrich et al. reported significantly lower maternal CST in preeclamptic compared with healthy pregnancies, with no difference between early- and late-onset disease, and showed that adding CST improved logistic-regression modelling of preeclampsia occurrence [11]. Similarly, Owolabi and Kasso documented reduced CST levels in preeclampsia and an inverse association with disease severity [12,14]. By contrast, Tüten et al. observed higher CST concentrations in preeclamptic women [13]. At the placental level, Bralewska et al. demonstrated higher chromogranin A (CgA) gene expression but lower CST protein levels in preeclamptic placentas, suggesting impaired post-translational processing of CgA to CST rather than reduced precursor availability [20]. A recent narrative review by Bralewska et al. [21] synthesised these clinical and experimental observations and concluded that, although the direction of change in maternal CST in preeclampsia is not yet uniformly established, the balance of evidence is compatible with reduced bioavailable CST in the preeclamptic milieu. Our finding of a physiological decline in CST across normotensive pregnancy provides a longitudinal reference against which these cross-sectional preeclampsia data can be interpreted, and indicates that the absolute concentration alone may be less informative than its trajectory.

Regarding maternal metabolic adaptation, Vanli Tonyali et al. found that women with gestational diabetes mellitus (GDM) had lower circulating CST concentrations than healthy pregnant controls, irrespective of the treatment approach used [22]. Our observation that a greater first-to-third-trimester decline in CST is associated with higher third-trimester insulin concentrations is directionally consistent with these data and extends them by capturing the within-individual change rather than a single late-gestation snapshot. Considered together, the existing cross-sectional human data and our longitudinal findings converge on the concept that CST tracks the metabolic and haemodynamic transition of pregnancy, but the magnitude and clinical relevance of these changes require confirmation in larger and more diverse cohorts.

### 4.3. Hypothesised Mechanisms

The mechanisms underlying the gestational CST trajectory observed here remain to be established, and the following considerations are explicitly hypothesis-generating rather than inferred from direct mechanistic data in our cohort.

First, the elevation of muscle sympathetic nerve activity observed during normotensive pregnancy, particularly in late gestation, may exceed the rate of CST replenishment, leading to a net decline in circulating peptide [23,24,25]. Under this hypothesis, the late-gestation reduction in CST reflects increased utilisation of an endogenous sympathoinhibitor rather than diminished synthesis. Whether such a mechanism operates in vivo cannot be determined from our data and would require parallel measurement of CgA, CST and direct sympathetic indices.

Second, reduced peripheral CST may interact with the established attenuation of neurovascular transduction during pregnancy, in which vascular resistance per unit of sympathetic outflow is markedly reduced [26,27,28]. Recent RAAS-focused evidence indicates that normal pregnancy is characterized by reduced vascular sensitivity to angiotensin II despite activation of the renin–angiotensin–aldosterone system, while recent human data link circulating CST with ambulatory blood pressure and arterial stiffness in primary hypertension. These observations support a plausible, but still unproven, role of CST as one of several modulators of Ang II-related vascular adaptation during pregnancy [10,29]. Our positive cross-sectional correlation between third-trimester CST and systolic blood pressure, in the absence of an association between Δ CST and Δ blood pressure, is consistent with CST acting as one of several modulators rather than a dominant determinant of gestational blood-pressure regulation.

Third, the inverse association between Δ CST and third-trimester insulin may reflect the documented effects of CST on hepatic insulin sensitivity, glucose uptake and macrophage-mediated inflammation in non-pregnant models [30,31,32]. A physiological reduction of CST could plausibly support the controlled insulin resistance of late pregnancy [31], whereas an exaggerated decline might shift the balance toward maladaptive insulin resistance, as suggested by lower CST in GDM [22]. This framework remains speculative in pregnancy: it is supported by mechanistic data from rodent and cell-line studies but has not been demonstrated in human gestation.

Fourth, placental contributions to circulating CST cannot be excluded. Bralewska et al. showed in HTR-8/SVneo and BeWo trophoblast cell lines that a preeclamptic-like environment reduces CgA-derived CST protein, implicating altered trophoblastic processing rather than transcription as a candidate mechanism [20]. Whether analogous, milder shifts in placental CST handling contribute to the physiological decline observed in normotensive pregnancy is unknown.

Finally, additional contributors that warrant systematic evaluation include maternal adiposity and low-grade inflammation [33], CST genotype with potential functional effects [34], and pre-analytical factors such as sampling time, fasting status and concurrent medications. The minority of participants showing a third-trimester increase in CST is best regarded as a metabolically modulated subgroup rather than evidence against the average downward trend; stratified analyses by body mass index and indices of insulin resistance, with concurrent inflammatory profiling and CST genotyping, are required to disentangle these trajectories.

### 4.4. Clinical Implications

The clinical implications of the present findings are preliminary and should be framed as hypothesis-generating. The demonstration that CST follows a reproducible longitudinal pattern in normotensive pregnancy supports its further evaluation as a candidate biomarker of maternal cardiometabolic adaptation, but does not, on the current evidence, justify its use in clinical decision-making.

Three avenues appear most promising. First, longitudinal CST trajectories—rather than single absolute values—may add information to existing risk-stratification tools for hypertensive disorders of pregnancy when assessed alongside established haemodynamic indices such as cardiac output and systemic vascular resistance [35]. This concept is consistent with recent reviews proposing CST as a potential adjunctive marker in preeclampsia, while emphasising that its diagnostic and prognostic performance has not yet been validated [21]. Second, the association between Δ CST and third-trimester insulin warrants prospective evaluation in cohorts at risk of GDM, in light of the lower CST levels reported in established GDM [22]. Third, given the convergent proangiogenic, antithrombotic and anti-inflammatory properties attributed to CST [36,37,38,39] and to low-dose acetylsalicylic acid in women at high risk of preeclampsia [40], a possible interaction between endogenous CST and aspirin pharmacodynamics is conceptually plausible and merits dedicated study, though no direct evidence is currently available.

In summary, the present data position catestatin as a measurable, dynamically regulated peptide that mirrors aspects of gestational cardiometabolic physiology. Its translation into a clinically useful biomarker will require adequately powered, multi-centre longitudinal studies in ethnically diverse populations, paired with concurrent measurement of haemodynamic and metabolic indices and with mechanistic work clarifying placental and systemic determinants of circulating CST.

### 4.5. Limitations

Several limitations should be acknowledged. First, the relatively small sample size and single-centre, exclusively Caucasian cohort limit generalisability. Second, CST was assessed at only two time points; a denser longitudinal design would more precisely characterise the trajectory and any inflection between the early peak and late-gestation nadir. Third, in the absence of pregnancy-specific reference intervals, interpretation of absolute CST concentrations remains constrained. Fourth, only normotensive women without comorbidities were included; whether the observed pattern is preserved, exaggerated or reversed in women at high risk of preeclampsia or GDM, and whether CST exhibits synergy with low-dose acetylsalicylic acid in such women [36,40], cannot be inferred from the present data. Finally, diet and habitual physical activity were not systematically captured, raising the possibility of residual confounding.

## 5. Conclusions

Serum catestatin concentrations declined significantly from the first to the third trimester in initially low-risk singleton pregnancies. Third-trimester catestatin was positively associated with systolic blood pressure, while longitudinal changes in catestatin were related to third-trimester insulin concentrations. These findings suggest that catestatin may reflect selected hemodynamic and metabolic adaptations during pregnancy, although its clinical relevance remains uncertain. Larger longitudinal studies, including women with hypertensive disorders of pregnancy and gestational diabetes, are needed to clarify its predictive value and underlying mechanisms.

## Figures and Tables

**Table 1:** Maternal clinical, biochemical, and uterine artery Doppler characteristics of the study cohort.

Characteristic	Pregnant Women (n = 50)
Gestational age at first-trimester assessment, weeks	11–13 + 6/7
Gestational age at third-trimester follow-up, weeks	30–41 + 5/7
Maternal age, years	31.6 ± 4.2
Gravidity, n (%)	
Primipara	26 (52%)
Second	13 (26%)
Third or more	11 (22%)
History of miscarriage, n (%)	5 (10%)
IVF, n (%)	3 (6%)
Body mass index, kg/m^2^	22.7 [21.1–25.3]
Smoker, n (%)	15 (30%)
Office blood pressure	
SBP, mmHg	124.8 ± 15.3
DBP, mmHg	79.6 ± 10.4
Hemoglobin, g/L	115.2 ± 8.4
Fasting blood glucose, mmol/L	4.3 [4.0–4.5]
Cholesterol, mmol/L	7.4 ± 1.3
LDL, mmol/L	4.2 ± 1.2
HDL, mmol/L	1.9 [1.7–2.2]
Triglycerides, mmol/L	2.8 [2.2–3.3]
Mean uterine artery PI (Left)	1.7 ± 0.6
Mean uterine artery PI (Right)	1.6 ± 0.6
Abnormal uterine artery PI, n (%)	7 (14%)

Abbreviations: SBP: systolic blood pressure; DBP: diastolic blood pressure; LDL: low-density lipoprotein; HDL: high density lipoprotein; CV: cardiovascular. Data presented as mean ± SD, median (IQR) or n (%), as appropriate. Gestational age is expressed as weeks+days.

**Table 2:** Univariate correlation analysis of factors associated with Δ Catestatin.

Parameter	r—Correlation Coefficient *	*p*
Systolic BP (3rd trimester)	0.202	0.159
Δ Systolic BP **	0.113	0.441
Insulin (3rd trimester)	0.341	0.015
Δ Insulin	0.006	0.978
Age	−0.231	0.106

* Spearman’s rank correlation coefficient ** Δ Systolic BP = Systolic BP 3rd trimester—Systolic BP 1st trimester. Abbreviations: BP: blood pressure.

**Table 3:** Multiple linear regression analysis of factors associated with Δ Catestatin.

Parameter	r^2^	Β	SE	*p*
Δ Systolic BP *	0.123	0.239	0.170	0.166
Creatinine (3rd trimester)		−0.010	0.303	0.974
Insulin (3rd trimester)		0.041	0.019	0.037
GDM		−10.48	8.24	0.201

* Δ Systolic BP = Systolic BP 3rd trimester—Systolic BP 1st trimester. Abbreviations: BP: blood pressure; GDM: gestational diabetes mellitus.

**Table 4:** Multiple linear regression analysis of factors associated with serum catestatin levels in 3rd trimester.

Parameter	r^2^	Β	SE	*p*
Systolic BP (3rd trimester)	0.154	0.175	0.062	0.007
Age		0.139	0.230	0.549
Insulin (3rd trimester)		0.002	0.007	0.745
Creatinine (3rd trimester)		0.021	0.112	0.849

Abbreviations: BP: blood pressure.

## Data Availability

The data presented in this study are available upon request to the corresponding author. The data are not publicly available because some of the data sets will be used for further research.

## Abbreviations

The following abbreviations are used in this manuscript:

AI	Artificial intelligence
ASA	Acetylsalicylic acid
BMI	Body mass index
BP	Blood pressure
β-hCG/ß-hCG	Beta-human chorionic gonadotropin
CgA	Chromogranin A
CI	Confidence interval
CRL	Crown-rump length
CST	Catestatin
CV	Cardiovascular
DBP	Diastolic blood pressure
EIA	Enzyme immunoassay
ER	Endoplasmic reticulum
GABA	Gamma-aminobutyric acid
GDM	Gestational diabetes mellitus
HDL	High-density lipoprotein
HOMA-IR	Homeostatic Model Assessment of Insulin Resistance
HRV	Heart rate variability
IQR	Interquartile range
IVF	In vitro fertilization
LDL	Low-density lipoprotein
MSNA	Muscle sympathetic nerve activity
NO	Nitric oxide
PAPP-A	Pregnancy-associated plasma protein A
PI	Pulsatility index
RVLM	Rostral ventrolateral medulla
SBP	Systolic blood pressure
SD	Standard deviation
SE	Standard error
SNA	Sympathetic nerve activity
SNS	Sympathetic nervous system
SPSS	Statistical Package for the Social Sciences
VIF	Variance inflation factor
α	Type I error rate/significance level
β/Β	Regression coefficient
Δ	Change/difference between measurements
n	Number of participants or observations
*p*	Probability value/statistical significance value
r	Correlation coefficient
r^2^/r^2^	Coefficient of determination
